# Short-Term Side Effects after Radioiodine Treatment in Patients with Differentiated Thyroid Cancer

**DOI:** 10.1155/2016/4376720

**Published:** 2016-02-17

**Authors:** Liyan Lu, Fengling Shan, Wenbin Li, Hankui Lu

**Affiliations:** ^1^Department of Nuclear Medicine, Shanghai Jiao Tong University Affiliated Sixth People's Hospital, No. 600 Yi Shan Road, Shanghai 200233, China; ^2^Department of Radiology, Shanghai Jiao Tong University Affiliated Sixth People's Hospital, No. 600 Yi Shan Road, Shanghai 200233, China

## Abstract

*Objectives*. I-131 therapy for differentiated thyroid cancer (DTC) could induce adverse effects. The purpose of this study was to report and analyze symptoms after I-131 treatment within the hospitalization and present relevant medical intervention.* Methods*. I-131 doses ranging from 3.7 to 9.25 GBq (100–250 mCi) were administrated for thyroid remnant ablation or treating DTC metastases. 117 patients with DTC for I-131 therapy were monitored through the video and intercommunicating with standardized questionnaire at different time points after I-131 oral administration. Adverse effects were recorded and relevant clinical factors were analyzed.* Results*. Among all the 117 patients, 55 cases complained of neck's pain or swelling and 79 cases presented with gastrointestinal symptoms. Pain or swelling of salivary gland occurred in 15 patients, headache and vertigo in 10, insomnia in 9, vocal cord paralysis in 6, fatigue or general malaise in 6, and foreign body sensation in 5. Body numbness and urinary symptoms were observed in only 1 case, respectively. Those side effects were related with sex, pre-I-131 treatment TSH levels, frequency of I-131 therapy, and lymph node metastases.* Conclusions*. Short-term side effects after I-131 therapy for DTC patients varied individually; severe symptoms were not uncommon but generally did not need emergent medical intervention.

## 1. Introduction

Radioactive iodine therapy with I-131 has been used in the management of differentiated thyroid cancer (DTC) for the ablation of residual thyroid or treatment of its metastases since 1946 [1]. Much concerns have been paid to long-term side effects and risks of high dose I-131 [2]. However, short-term symptoms especially the serious acute complications associated with I-131 therapy, though extremely common, have been poorly documented. In many regions, DTC patients have been treated as the outpatients rather than inpatients. In outpatients mode, radioactivity pollution prevention outweighs medical concerns and instant symptoms after I-131 treatment have not been systematically reported [3–5].

In our institute, however, we have kept DTC patients in hospitalization for a week not only for restricting patients from public for radiation protection but also for careful monitoring in case of any severe symptoms which may need proper intervention during hospitalization. In the past, we have noticed a large percentage of DTC patients complaining of uncomfortable symptoms such as pain or swelling in the neck and parotid region, dryness of mouth, altered taste, and difficulty in swallowing as have been described in the literature [3–9]. However, they did not provide any data concerning those symptoms and relevant factors that may compound those situations.

The aim of this study was to investigate the prevalence of short-term symptoms in patients with DTC who received a total or subtotal thyroidectomy followed by I-131 therapy. We reported our experience in order to highlight these problems and emphasize the importance of early recognition to the problems.

## 2. Materials and Methods

### 2.1. Ethics Statements

The study was approved by the Institutional Review Board of Shanghai Jiao Tong University Affiliated Sixth People's Hospital and it was performed in accordance with the Declaration of Helsinki. All patients signed informed consent.

### 2.2. Patients Data

117 patients (74 females, 43 males; mean age 41.6 ± 13.6 years, range 13–73 years) with DTC (115 papillary, 2 follicular) who underwent total or subtotal thyroidectomy and subsequently received I-131 ablation and treatment in our department were included in the present study. Lymph node metastases were present in 25 patients and 20 patients had distant metastases.

82 patients were treated once for thyroid remnant ablation while others were treated twice or more for DTC metastases in which 23 patients were treated twice, 7 three times, 2 four times, and 3 five times. Cumulative activity ranged from 100 to 1400 mCi (mean 192.7 mCi, standard deviation 176.3 mCi).

### 2.3. Preparation

Before I-131 therapy, all patients followed a standard protocol including the lower iodine diet and avoiding radiological contrast, withdrawing thyroid hormone medication 3-4 weeks to stimulate serum TSH values to high levels. One day ahead of the I-131 therapy, only five patients failed to achieve the serum TSH levels > 30 mIU/L. Other lab tests and medical examinations consisted of the following: serum FT3, FT4, Tg, TgAb, PTH, neck ultrasonography, neck and chest X-CT scan, and MRI.

### 2.4. Administration

After oral I-131 administration, the patients were admitted and restricted in the purpose-built wards with specialized disposal facilities for radioactive waste over a seven-day period. The dose of 3.7 GBq (100 mCi) of I-131 was used to ablate the thyroid remnants, 5.5 GBq (150 mCi) for patients with lymph node metastasis and 7.4 GBq–9.25 GBq (200–250 mCi) for distant metastases.

I-131 whole body scan (I-131-WBS) followed by I-131 SPECT/CT imaging was performed 5 days after 131-I therapy.

### 2.5. Inpatient Monitoring of Adverse Events and Relevant Management

All patients were carefully investigated during hospitalization with video-monitoring with their consents. They were asked and answered the questions, through intertelecommunication concerning pain and swelling difficulties in the neck or salivary glands, dry mouth, gastrointestinal discomforts, vocal cord paralysis, menstrual irregularities, headache and vertigo, insomnia, fatigue or general malaise, and foreign body sensation. Patients were especially asked whether they had previously experienced these uncomfortable symptoms and none gave a history of preexistence of these symptoms. The monitoring time points were 2 h, 24 h, 48 h, 72 h, and 96 h after I-131 oral administration.  Table 1 listed our standardized questions and proper advice and management given to the patients [8].

### 2.6. Statistical Analysis

Documented results are presented as mean ± standard deviation and median value when appropriate. The relationship between RAI administration activity and the development of symptoms was investigated using logistic regression analysis, which was estimated separately for each symptom using maximum likelihood.

## 3. Results

**Figure 1:**
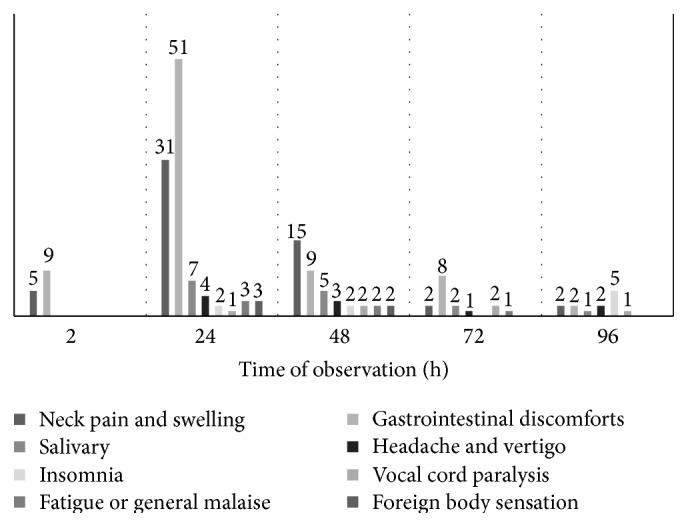
Comparison of short-term discomforts (except body numbness and urinary disorder) with different time points of observation.

**Figure 2:**
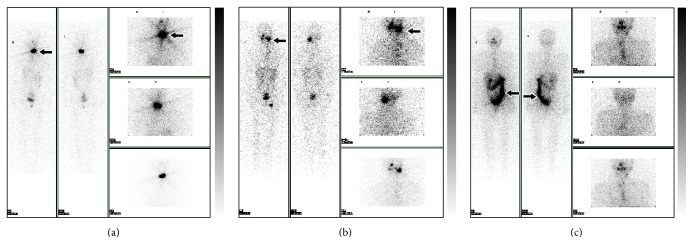
^131^I whole body scan. (a) ^131^I uptake in the thyroid region. (b) ^131^I uptake in the salivary glands. (c) ^131^I uptake in the intestine.

Among all the 117 patients, 55 patients (55/117, 47.0%) complained of neck's pain or swelling after I-131 treatment. Of these 55 patients, 5 patients had pain or swelling of neck at 2 h, 31 at 24 h, 15 at 48 h, 2 at 72 h, and 2 at 96 h, respectively. 51 patients (51/55, 92.8%) developed pain or swelling of neck within 48 h of treatment, mainly from 24 h to 48 h (Figure 1), only 4 patients (4/55, 7.2%) developed the symptom after 48 h, and the symptom did not become apparent until 72 h after therapy. And 55 patients accumulated a great deal of I-131 activities in the thyroid region throughout I-131 whole body scan (Figure 2(a)).

Gastrointestinal discomforts were reported in 79 of 117 cases (79/117, 67.5%), including 9 cases at 2 h, 51 at 24 h, 9 at 48 h, 8 at 72 h, and 2 at 96 h. Three patients were reported to have vomiting, and 8 patients had astriction or diarrhea. The complaints usually occurred within 48 h and as early as 2 h. Mostly, nausea with vomiting occurred at 24 h (Figure 1). However, astriction or diarrhea happened at random time ranging from 24 h to 96 h. 79 patients accumulated a great deal of I-131 activities in gastrointestinal tract when given I-131 whole body scan (Figure 2(c)).

Symptoms or signs of the salivary gland occurred in 15 patients with pain in 12 patients, swelling in 12, and dry mouth in 4. The onset of the pain or swelling of salivary and dry mouth focused at 24 h–48 h (Figure 1), and these symptoms were mild and easily controlled. Patients with uncomfortable symptoms in salivary gland accumulated more I-131 in this region throughout I-131 whole body scan (Figure 2(b)).

10 patients (10/117, 8.5%) were reported to have headache and vertigo; 9 patients (9/117, 7.7%) were reported to have insomnia. Vocal cord paralysis occurred in 6 patients (6/117, 5.1%), fatigue or general malaise in 6 patients (6/117, 5.1%), and foreign body sensation in 5 patients (5/117, 4.3%). Urinary symptoms and body numbness occurred in 1 patient at 96 h, respectively. In addition, 3 females were in their menstrual period during I-131 treatment. Insomnia, headache and vertigo, vocal cord paralysis, fatigue or general malaise, foreign body sensation, body numbness, and urinary symptoms happened without significant regularity (from 2 h to 96 h) (Figure 1); thus we failed to make any significant conclusions.

The association of I-131 therapy with short-term symptoms was assessed using logistic regression analysis. Pain or swelling of neck was related to sex, pre-I-131 treatment TSH level, and episode of treatment. Female patients (41/55, 74.5%) were more likely to develop neck pain or swelling than male (14/55, 25.5%) (*P* = 0.004). Another compounding factor was low pre-I-131 treatment TSH level with relatively large thyroid remnant. Pain or swelling of neck was significantly less frequent in repeated I-131 therapy for DTC metastases.

Gastrointestinal discomforts were also found to correlate with pre-I-131 treatment TSH levels but in different direction. DTC patients with high pre-I-131 treatment TSH level are prone to developing severe gastrointestinal symptoms. Pain or swelling of salivary and dry mouth were related to the sex, episode of therapy, or lymph node involvements. More female patients (14/15, 93.3%) developed salivary gland dysfunction than male (1/15, 6.7%) (*P* = 0.04). Other compounding factors were repeated I-131 therapy and more node involvement. While there was no statistically significant difference between the patients who received high rational dose and those who received low, the relationship between age and all of those symptoms is not of significance (Table 2).

## 4. Discussion

Van Nostrand et al. [10] reported their preliminary experience of immediate (during hospitalization) and intermediate (after discharge up to 3 months) side effects resulting from I-131 treatments based on doses calculated by BEL dosimetric approach and described side effects in detail. However, the patients they elected all had metastatic lesions and they mainly studied the relationship between rational doses and side effects. Further investigation in the field has been scarce.

It has been known for many years that short-term symptoms associated with high dose I-131 treatment for DTC are fairly common [2, 5, 11, 12]. However, neck's pain or swelling complaint was less common in those studies than in ours. Benua et al. [13] reported that only 9 of 122 patients developed neck pain or swelling, while Van Nostrand et al. [10] reported about 20% patients that presented with the complaints. The higher frequency of neck's pain or swelling in our study may probably be attributed to the pretty large thyroid remnant and more careful monitoring than in those studies. In the past, though not reported, we had two patients that developed severe short of breath and had to resort to tracheal intubation. Lastly, we generally discourage patients to use dexamethasone for relieving neck pain or swelling in light of mild symptom in general and avoidance of negative impact from the dexamethasone.

The most common symptoms after I-131 treatment are gastrointestinal discomforts, as previously reported in other studies [2, 4, 10, 14]. Salivary gland swelling, pain, and dysfunction have been the most notable symptoms due to their I-131 uptake and discretion [2, 4, 8, 9, 15]. Dry mouth is a complication which develops late after salivary gland swelling and dysfunction which might be even more frequent when patients are discharged from the hospital. More disturbing to the patients have been the nausea and vomiting which usually occur within 36 h after I-131 administration. Worth notice is that some patients came up with nausea as early as 2 h. However, typical cases became obvious at 24 h or later and lasted for 2 days.

As mentioned above, pain or swelling of neck and gastrointestinal discomforts were found to correlate with pre-I-131 treatment TSH levels but in different direction. DTC patients were prone to developing pain or swelling of neck with low pretreatment TSH levels while severe gastrointestinal symptoms were with high pretreatment TSH levels. The low pretreatment TSH levels may indicate the relatively larger residual thyroid tissue; the higher concentration of radioiodine in thyroid might cause pain and swelling of neck. Meanwhile high pre-I-131 treatment TSH levels indicate smaller thyroid remnant; therefore we suggest that I-131 uptake in gastrointestinal tract may increase, which induces the gastrointestinal disorder.

What puzzled us is that contrary to previous study [10], the total number of patients in our study is sufficient to demonstrate any relationship between various symptoms and some independent variables (Table 2). It is interesting that some symptoms such as pain or swelling of neck and gastrointestinal discomforts are related to sex, which means that female patients are more prone to I-131 side effects rather than males. But further studies will be needed to confirm this phenomenon.

In our study, body numbness in one patient was probably caused by direct I-131 which exerted further damage to the parathyroid. Vocal cord paralysis as a side effect was reported in some studies [16–20]. After total thyroidectomy, most of those patients already experienced some degree of vocal cord paralysis. Exposure to I-131 causes neck soft tissue edema which leads to vocal cord temporal damage and radiation thyroiditis indirectly induces bilateral nerve injury. Most cases slowly recovered from vocal cord paralysis.

We also noticed that 7.7% of patients developed insomnia during hospitalization. Those patients may worry much about their conditions while anxiously waiting for the posttreatment I-131 whole body scan.

Other rare symptoms were also found in our study. Ten patients developed headache and vertigo, 6 patients developed fatigue or general malaise, and 5 patients complained of foreign body sensation. Only one patient in our study complained of urinary disorder. Temporary amenorrhea/oligomenorrhea may occur in as many as 20–27% of women [2]; however, only three females developed menstrual irregularities in their menstrual period in this study.

There are two major limitations in our study. First, data about short-term uncomfortable symptoms were collected through video monitoring, some symptoms were verified by I-131 whole body scan, and the others were lack of objective evidence to verify whether these symptoms directly react to I-131 treatment. Second, it is difficult to obtain any relationship to independent variables because of the small sample size of body numbness, urinary disorder, and menstrual irregularities. So further investigations should be needed to resolve the two major problems.

## Figures and Tables

**Table 1:** Standardized questions and proper advice and management.

Questions	Proper advice or care
Do you feel any neck pain and (or) swelling difficulty or shortness of breath?	Use cold applications and/or prednisone
Do you have dry mouth and/or salivary swelling?	Drink more water
Do you feel any gastrointestinal discomfort and have nausea and/or vomiting?	Take gastric menses
Do you have headache or vertigo?	No intervention
Do you feel fatigue or general malaise?	No intervention
Do you have uneasiness or insomnia?	Sometimes take Valium
Do you have vocal cord paralysis or worsening paralysis?	Neurotrophic medicine
Do you feel body numbness worsening?	Calcium tablets
Do you have urinary symptom?	No intervention
Are you on menstrual period now? Do you have dysmenorrhea?	No intervention

**Table 2:** Logistic regression analysis with independent variables for *P* value < 0.05 was considered in which symptoms were related with the clinical signs.

Independent variables	Pain or swelling of neck	Gastrointestinal discomforts	Salivary gland dysfunction
Age			
Sex	0.004		0.04
TSH level	0.017	0.007	
Lymph node metastases			0.00998
Distant metastases			
Episode of therapy	0		0.0088
Cumulative activity			
